# Plant eIF4E isoforms as factors of susceptibility and resistance to potyviruses

**DOI:** 10.3389/fpls.2023.1041868

**Published:** 2023-02-10

**Authors:** Nikolay Zlobin, Vasiliy Taranov

**Affiliations:** Laboratory of plant tress tolerance, All-Russia Research Institute of Agricultural Biotechnology, Moscow, Russia

**Keywords:** plant resistance, recessive resistance, susceptibility gene, potyvirus, eIF4E isoform

## Abstract

Potyviruses are the largest group of plant-infecting RNA viruses that affect a wide range of crop plants. Plant resistance genes against potyviruses are often recessive and encode translation initiation factors eIF4E. The inability of potyviruses to use plant eIF4E factors leads to the development of resistance through a loss-of-susceptibility mechanism. Plants have a small family of eIF4E genes that encode several isoforms with distinct but overlapping functions in cell metabolism. Potyviruses use distinct eIF4E isoforms as susceptibility factors in different plants. The role of different members of the plant eIF4E family in the interaction with a given potyvirus could differ drastically. An interplay exists between different members of the eIF4E family in the context of plant–potyvirus interactions, allowing different eIF4E isoforms to modulate each other’s availability as susceptibility factors for the virus. In this review, possible molecular mechanisms underlying this interaction are discussed, and approaches to identify the eIF4E isoform that plays a major role in the plant–potyvirus interaction are suggested. The final section of the review discusses how knowledge about the interaction between different eIF4E isoforms can be used to develop plants with durable resistance to potyviruses.

## 1 Introduction

Plant viruses are microscopic organisms with a small genome that typically encodes a dozen proteins. It is insufficient to complete the different stages of the viral life cycle, i.e., multiplication within cells, cell-to-cell movement, and long-distance systemic movement in the plant. To accomplish this, viruses have to use various host proteins. These proteins function as susceptibility factors that allow a compatible interaction between the virus and the plant. In theory, the unavailability of even one of these factors for the virus in the plant cell will make the interaction incompatible and lead to resistance. This type of resistance, known as loss of susceptibility, has been observed in various plant–pathogen systems, mainly for obligate biotrophic pathogens such as viruses (Pavan et al., 2010). Unlike more well-recognized resistance mechanisms that rely on R-genes to recognize pathogen effectors and mount defense responses, resistance through loss of susceptibility is generally characterized by increased durability and spectrum (Pavan et al., 2010; van Schie and Takken, 2014).

Because loss of susceptibility is based on the nonavailability of a certain cell factor to the pathogen, this resistance is inherited recessively. The proportion of recessively inherited resistance genes to plant viruses is extraordinarily high (Truniger and Aranda, 2009). This is especially true for potyviruses, the largest group of plant-infecting RNA viruses that cause severe crop losses worldwide (Yang et al., 2021). Less than half of the genes that confer resistance to these viruses are inherited recessively (Provvidenti and Hampton, 1992; Truniger and Aranda, 2009). The potential diversity of the host genes that encode susceptibility factors in plant–potyvirus interactions is enormous. The ability to promote potyviral infection was shown for numerous cellular proteins, from enzymes (Beffa et al., 1996; Ouibrahim et al., 2014; Poque et al., 2015) and carrier proteins (Park et al., 2017; Wu et al., 2020; Soler-Garzón et al., 2021a) to transcription factors (Endres et al., 2010; Soler-Garzón et al., 2021b), as well as many other proteins with diverse functions (Hofius et al., 2007; Castelló et al., 2010; Hafrén et al., 2010; Zhu et al., 2014; De et al., 2020; Bruckner et al., 2022; Zuo et al., 2022). However, the study of the genes that confer natural recessive resistance to potyviruses in various crop plants revealed that the molecular basis of this resistance is surprisingly similar in most cases. In different plants, natural recessive resistance to numerous potyviruses was shown to rely on the polymorphism of just a single type of cell factor, i.e., the translation initiation factor eIF4E. In some of these plants, several independently discovered recessive resistance genes were eventually shown to encode the same eIF4E factor (**Table 1**). Therefore, eIF4E-mediated resistance to potyviruses has evolved numerous times in systematically distinct plant species independently. Moreover, eIF4E-mediated resistance to various groups of viruses other than *Potyviruses* (*Bymoviruses*, *Potexviruses*, *Tritimoviruses*, *Ipomoviruses*, *Carmoviruses*, *Carlaviruses*, and *Cucumoviruses*) was discovered in nature (Kanyuka et al., 2005; Stein et al., 2005; Nieto et al., 2006; Nieto et al., 2007) or artificially generated (Yoshii et al., 2004; Keima et al., 2017; Rodríguez-Hernández et al., 2012; Rupp et al., 2019; Chen et al., 2022b).

**Table 1:** Recessive genes conferring resistance against potyviruses to crop plants.

Plant	Gene	Virus(es)^(1)^	Protein	Type of polymorphism	References
eIF4E factors					
Pepper	*pvr1* *pvr2*	PVY, TEV, PTV, PepMoV, PVMV^(2)^, ChiVMV^(2)^	eIF4E	aa changes	Ruffel et al., 2002; Kang et al., 2005; Ruffel et al., 2006; Hwang et al., 2009; Moury et al., 2014a; Moury et al., 2014b
	*pvr6* ^(3)^	PVMV, ChiVMV	eIF(iso)4E	large deletion	Ruffel et al., 2006; Hwang et al., 2009; Rubio et al., 2009
Tomato	*pot-1*	PVY, TEV, PepMoV	eIF4E	aa changes	Ruffel et al., 2005; Gauffier et al., 2016; Lebaron et al., 2016
Chinese cabbage	*retr01* ^(4)^ *retr02* ^(4)^	TuMV	eIF(iso)4E (eIF(iso)4E.a)	miss-splicing mutant	Rusholme et al., 2007; Qian et al., 2013; Nellist et al., 2014
Pea	*cyv-2* *wlv* *sbm1* *sbm4*	PSbMV, BYMV, ClYVV	eIF4E	aa changes	Provvidenti and Alconero, 1988a, 1988b; Gao et al., 2004; Bruun-Rasmussen et al., 2007; Andrade et al., 2009
Common bean	*bc-3* *desc* *cyv*	ClYVV, BCMV, BCMNV	eIF4E	aa changes	Naderpour et al., 2010; Hart and Griffiths, 2013, 2014
Lettuce	*mo1*	LMV	eIF4E	aa changes	Nicaise et al., 2003
Tobacco	*va*	PVY, TVMV	eIF4E (eIF4E-1/eIF4E1-S)	large deletion/ miss-splicing mutant	Julio et al., 2015; Lin et al., 2021
Likely eIF4E factors					
Watermelon	*zym*	ZYMV	eIF4E	aa changes	Ling et al., 2009
Unlikely eIF4E factors					
Pea	*sbm2* *bcm* *cyv1* *mo pmv*	PSbMV, BYMV, ClYVV, WMV	ND	–	Provvidenti and Hampton, 1991; Choi et al., 2012
Chinese cabbage	*trs*	TuMV	ND	–	Kim et al., 2013; Palukaitis and Kim, 2021
Melon	–	WMV	ND	–	Gonzalez-Ibeas et al., 2012
Non-eIF4E factors					
Mustard	*retr03*	TuMV	eIF2β	aa changes	Shopan et al., 2017
Common bean	*bc-2* ^(5)^	BCMV	Vps4 AAAC ATPase	frameshift deletion	Soler-Garzón et al., 2021a
Cucumber	*zym*	ZYMV	VPS4-like	aa changes	Amano et al., 2013

aa changes, amino acid changes; PVY, potato virus Y; TEV, tobacco etch virus; PepMoV, pepper mottle virus; PTV, Peru tomato mosaic virus (formerly Peru tomato virus); PVMV, pepper veinal mottle virus; ChiVMV, Chilli veinal mottle virus; TuMV, turnip mosaic virus; PSbMV, pea seed-borne mosaic virus; BYMV, bean yellow mosaic virus; ClYVV, clover yellow vein virus; BCMV, bean common mosaic virus; BCMNV, bean common mosaic necrosis virus; LMV, lettuce mosaic virus; TVMV, tobacco vein mottling virus; ZYMV, zucchini yellow mosaic virus; WMV, watermelon mosaic virus.

^(1)^Could differ for different alleles.

^(2)^In conjunction with certain alleles of the eIF(iso)4E isoform.

^(3)^In conjunction with certain alleles of the eIF4E1 isoform.

^(4)^Requires the presence of the dominant gene ConTR01 to provide robust broad-spectrum resistance.

^(5)^Requires the presence of either the bc-4 or bc-u gene to manifest resistance.

The recessive nature of eIF4E-mediated resistance implies the inability of viruses to use certain allelic variants of eIF4E as susceptibility factors to complete their life cycles within plants. This type of resistance can manifest on different levels, including the inhibition of virus multiplication within the cell (Deom et al., 1997; Murphy et al., 1998; Lellis et al., 2002; Sato et al., 2005), cell-to-cell movement (Arroyo et al., 1996; Sato et al., 2003; Gao et al., 2004), and long-distance and systemic movement (Acosta-Leal and Xiong, 2008; Contreras-Paredes et al., 2013). This suggests that eIF4Es have a wide range of functions during the viral life cycle, which is presumably one of the causes of their prevalence as susceptibility factors for different viruses.

eIF4E-mediated resistance is often durable and is not overcome by viruses in the field. Some recessive genes encoding eIF4E have been widely used in agriculture for over half a century and have not been broken down (van Schie and Takken, 2014). EIF4E-mediated resistance is also characterized by a broad spectrum, because a single *eIF4E* allele often provides resistance to different viral strains and isolates or even to different viruses (Rusholme et al., 2007; Andrade et al., 2009; Moury et al., 2014a; Gauffier et al., 2016). In contrast to many other resistance mechanisms mostly discovered in wild relatives of cultivated plants, eIF4E-mediated resistance occurs more frequently in domesticated plant populations than in wild ones (Hofinger et al., 2011; Konečná et al., 2014; Poulicard et al., 2016). This suggests that this type of resistance is particularly suited to cultivated plants and is associated with mild pleiotropic effects on crop growth and productivity. Together, these advantages have led to the widespread use of eIF4E-mediated resistance in agriculture.

## 2 Plants have a small family of eIF4E factors

eIF4E is one of the factors constituting the translation initiation complex. eIF4E binds to the 5′-cap of mRNA and eIF4G, leading to the formation of the eIF4F complex, which is recruited to the 40S ribosome subunit together with the other translation initiation factors. In addition to translation, eIF4E participates in other mRNA-related processes, in particular the transport of mRNA from the nucleus to the cytoplasm (Wang and Krishnaswamy, 2012).

**Figure 1:**
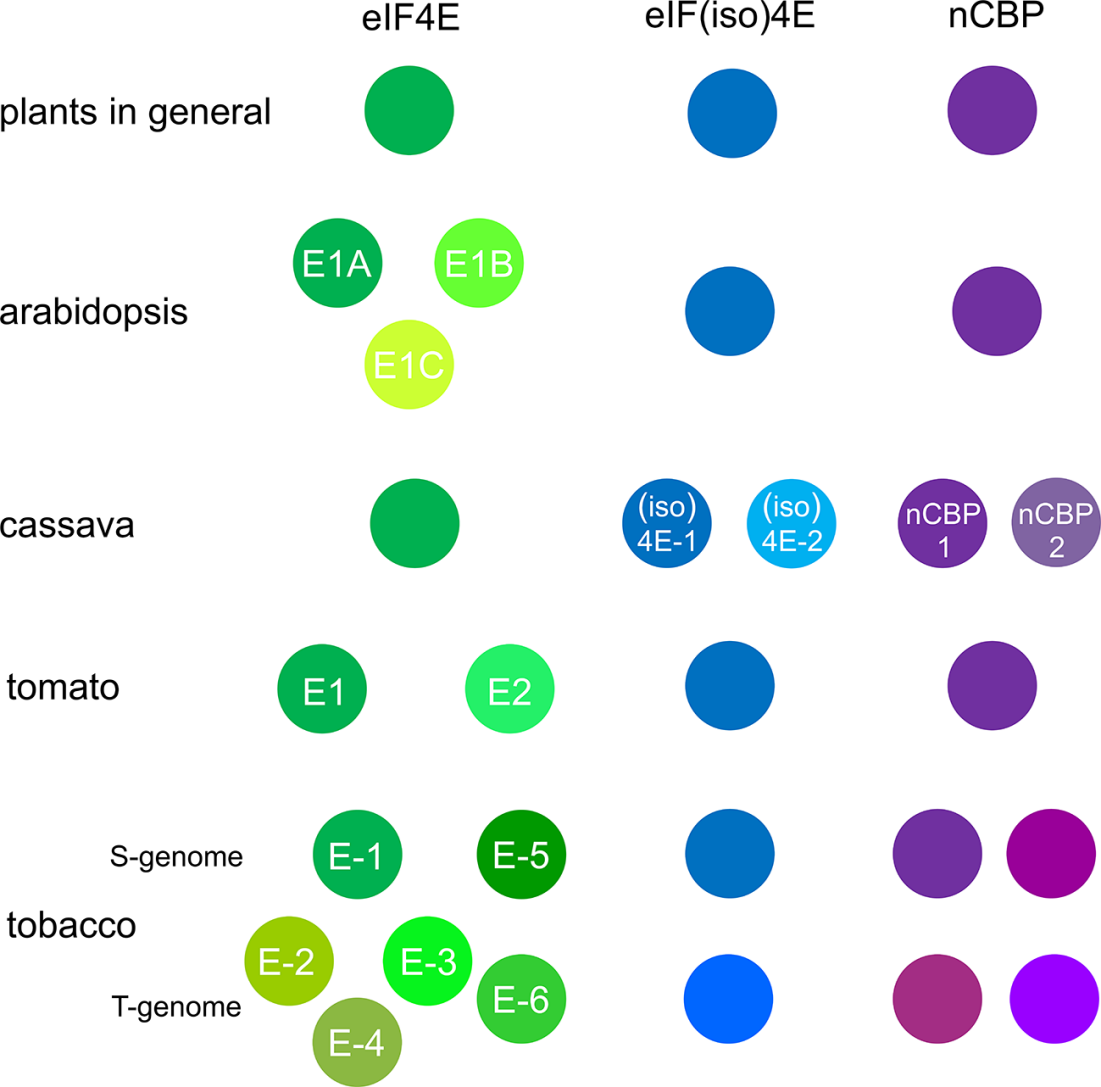
Plants contain several eIF4E isoforms. The names of some additional isoform members were as per those given by Patrick et al. (2014) for *Arabidopsis*, Gomez et al. (2019) for cassava, Piron et al. (2010) for tomato, and Michel et al. (2019) for tobacco.

In plants, there is not a single eIF4E factor; rather, they constitute a small family (**Figure 1**). In addition to eIF4E, flowering plants contain its isoform, which is known as eIF(iso)4E (Patrick and Browning, 2012). Although the members of this family have a relatively low level of sequence homology, they probably have a similar three-dimensional structure (Estevan et al., 2014) and overlapping functions. eIF4E1 inactivation in *Arabidopsis thaliana* was associated with mild pleiotropic effects under laboratory conditions; the resulting plants were smaller and had a 7-day delayed bolting and a slightly decreased seed yield (Sato et al., 2005; Bastet et al., 2018). The effects of eIF(iso)4E inactivation on *Arabidopsis* were even weaker, with only one study reporting some changes in root development (Duprat et al., 2002; Lellis et al., 2002; Sato et al., 2005; Martínez-Silva et al., 2012). *Arabidopsis* eIF4E1 was also shown to be involved in root growth regulation (Liu et al., 2022). In contrast, double knockout of both eIF4E1 and eIF(iso)4E in *Arabidopsis* was lethal (Callot and Gallois, 2014). *Arabidopsis* eIF4E1 or eIF(iso)4E inactivation decreased tolerance to low temperatures (Salazar-Díaz et al., 2021), while had no effect on elf18-induced resistance against the bacteria *Pseudomonas syringae* pv. *maculicola* (Wang et al., 2022). Cucumber plants with *eIF4E* knockout were viable and fertile, and pepper varieties containing inactivated eIF(iso)4E are used in agriculture (Ruffel et al., 2006; Chandrasekaran et al., 2016). In contrast, targeted inactivation of the eIF4E gene in barley leads to a yield penalty and to male sterility in melons (Hoffie et al., 2021; Pechar et al., 2022). eIF4E and eIF(iso)4E participate in the translation of various sets of mRNAs (Dinkova et al., 2011; Martínez-Silva et al., 2012). In *Nicotiana benthamiana*, eIF(iso)4E was suggested to be primarily associated with rough endoplasmic reticulum, whereas eIF4E was suggested to be primarily associated with free ribosomes (Beauchemin et al., 2007). The difference in eIF4E and eIF(iso)4E functions may be more pronounced in plants grown under stress conditions as opposed to the optimal growth conditions used in most studies (Martínez-Silva et al., 2012).

In addition to eIF4E and eIF(iso)4E, plants have another eIF4E family member known as nCBP (from novel cap-binding protein) (Ruud et al., 1998; Patrick and Browning, 2012). nCBP binds to the 5′-cap and interacts with eIF(iso)4G *in vitro* (Ruud et al., 1998; Kropiwnicka et al., 2015). Its functions in the context of plant growth and development have been barely studied (Christie and Igreja, 2021). In potatoes, *nCBP* knockdown causes deformation of young leaves (Chen et al., 2022a).

Additional eIF4E factors have been discovered in different plants (**Figure 1**) (Patrick and Browning, 2012; Patrick et al., 2014). In addition to eIF4E1, eIF(iso)4E, and nCBP, *Arabidopsis* plants have a locus that contains two other eIF4E genes *EIF4E1B* (also *EIF4E3*) and *EIF4E1C* (also *EIF4E2*) (Patrick et al., 2014). Although these factors have a high sequence homology to eIF4E1 (eIF4E1A), can bind m7GTP and eIF4G, and can initiate translation in yeast cells, they cannot compensate for the depletion of eIF4E1 and eIF(iso)4E in *Arabidopsis* (Patrick et al., 2014). The *Brassica rapa* genome comprises three genes encoding eIF4E and three genes encoding eIF(iso)4E, which are denoted by the letters a, b, and c, with one of the eIF4Es (eIF4E.a) being a pseudogene (Jenner et al., 2010; Nellist et al., 2014). Cassava has a single eIF4E gene but two eIF(iso)4E and two nCBP genes, all of which are expressed, albeit at significantly different levels (Shi et al., 2017; Gomez et al., 2019). In the genomes of different plants of the Solanaceae family, i.e., pepper, tomato, and potato, two homologous eIF4E variants, *eIF4E1* and *eIF4E2*, were reported (Piron et al., 2010; Lebaron et al., 2016; Lucioli et al., 2022). In tomato, simultaneous inactivation of *eIF4E1* and *eIF4E2* genes caused dwarfism, in contrast to the separate inactivation of *eIF4E1* or *eIF4E2* (Gauffier et al., 2016; Kumar et al., 2022); interestingly, the double knockout of *eIF(iso)4E* and *eIF4E2* in tomato was lethal (Bastet et al., 2017). Cultivated *Nicotiana tabacum*, which is an allotetraploid derived from the natural interspecific hybridization between *Nicotiana sylvestris* (S-genome) and *Nicotiana tomentosiformis* (T-genome), contains as many as 12 genes encoding eIF4Es, i.e., six eIF4E (eIF4E-1 to eIF4E-6), two eIF(iso)4E, and four nCBP (Julio et al., 2015; Michel et al., 2019). In addition, some tobacco varieties have additional copies of some *eIF4E* genes from *N. tomentosiformis* and even recombinants between these genes (Michel et al., 2019).

## 3 eIF4E isoforms as susceptibility factors for potyviruses

A molecular genetic analysis of recessive genes that determine potyvirus resistance in different crop plants revealed that most of them encode an eIF4E isoform, while some encode eIF(iso)4E (**Table 1**). Although different potyviruses use different eIF4E isoforms, resistance to certain potyvirus usually relies on the allelic composition of a single gene (**Table 1**). This suggests that specific potyvirus generally rely on a single eIF4E isoform as a susceptibility factor from several potentially available isoforms in the host plant.

In rare cases, natural eIF4E-mediated resistance is controlled by two recessive genes simultaneously. Pepper resistance to Chilli veinal mottle virus (ChiVMV) or pepper veinal mottle virus (PVMV) requires certain alleles of both *eIF4E1* and *eIF(iso)4E* (Ruffel et al., 2006; Hwang et al., 2009; Rubio et al., 2009). Surprisingly, these viruses use pepper eIF4E1 and eIF(iso)4E but not eIF4E1 and eIF4E2, which share more similar primary structures.

In plants containing several members of some eIF4E isoforms, their roles as susceptibility factors to potyviruses may differ remarkably. For example, tobacco contains as many as six variants of eIF4E inherited from both *N. sylvestris* and *N. tomentosiformis* (**Figure 1**) (Julio et al., 2015; Michel et al., 2019). Although all of these variants are classified as eIF4E, only one is a susceptibility factor for potyviruses potato virus Y (PVY), tobacco vein mottling virus (TVMV), and tobacco etch virus (TEV) (Rufty et al., 1989; Nicolas et al., 1997; Julio et al., 2015). This variant is inherited by tobacco from *N. sylvestris* and is known as eIF4E-1 (Michel et al., 2019) or eIF4E1-S (Takakura et al., 2018). Similarly, in *B. rapa*, the roles of the three eIF(iso)4E variants, i.e., eIF(iso)4E.a, b, and c, in turnip mosaic virus (TuMV) infection are also very different (Nellist et al., 2014).

Sometimes a single potyvirus uses different eIF4E isoforms in different plants. For example, TEV and lettuce mosaic virus LMV use eIF4E in pepper and lettuce (Ruffel et al., 2002; Nicaise et al., 2003) but rely on eIF(iso)4E in *Arabidopsis* (Lellis et al., 2002; Estevan et al., 2014). PVMV uses both eIF4E1 and eIF(iso)4E to infect pepper (Ruffel et al., 2006) but requires eIF4E2 to infect another Solanaceae family member, tomato (Moury et al., 2020).

### 3.1 Polymorphisms in the eIF4E isoform prevent its use by potyviruses

Currently, a large number of eIF4E allelic variants associated with resistance to potyviruses have been reported. The comparative analysis of these alleles from susceptible and resistant plants revealed that their amino acid sequences are generally highly similar. eIF4E alleles from resistant plants often differ from alleles from susceptible ones by only a few (generally 2–4) amino acid substitutions (Gao et al., 2004; Ruffel et al., 2005; Charron et al., 2008; Hart and Griffiths, 2013). They are mostly nonconserved, leading to considerable changes in the charge or hydrophobicity of the corresponding protein regions (Nicaise et al., 2003; Gao et al., 2004; Charron et al., 2008; Poulicard et al., 2016). These substitutions are located in the region of the eIF4E molecule that interacts directly with the potyviral protein VPg (Gao et al., 2004; Charron et al., 2008; Perez et al., 2012). This protein is covalently bound to the potyviral mRNA and mimics the 5′-cap of cellular mRNAs, thus allowing the translation of the potyviral polyprotein and providing some other eIF4E-mediated functions in the viral life cycle, such as cell-to-cell transport (Wang and Krishnaswamy, 2012; Tavert-Roudet et al., 2017). Nonconserved substitutions in eIF4E disrupt its binding to VPg (Kang et al., 2005; Charron et al., 2008), thus hindering viral multiplication and/or spread in the plant. If the plant only has resistant alleles of the eIF4E isoform, which the virus uses as a susceptibility factor, it is resistant to this virus.

### 3.2 The breakdown of eIF4E-mediated resistance is typically based on the re-interaction between VPg and eIF4E

Either under natural infectious conditions or after artificial inoculation, some plants with resistant eIF4E alleles in the homozygous state after viral inoculation eventually display disease symptoms (Ayme et al., 2006; Naderpour et al., 2010; Moury et al., 2014a; Moury et al., 2014b; Congdon et al., 2016; Lebaron et al., 2016). Only a small percentage of plants developed disease, and symptom appearance was significantly delayed. This indicates that potyviruses can overcome the loss of susceptibility mediated by polymorphisms in eIF4E, resulting in resistance breaking (RB). In potyvirus samples isolated from infected plants, point mutations that led to amino acid substitutions in viral proteins were found. Although RB could be achieved through mutations in various potyviral proteins (Hjulsager et al., 2002; Nakahara et al., 2010; Sorel et al., 2014) and possibly in the viral mRNA itself (Krause-Sakate et al., 2005), the most common way to overcome eIF4E-mediated resistance in different plant–potyvirus pathosystems is through single or multiple substitutions in the VPg protein (Borgstrøm and Johansen, 2001; Sato et al., 2003; Moury et al., 2004; Ayme et al., 2007; Bruun-Rasmussen et al., 2007; Perez et al., 2012; Moury et al., 2014a; Moury et al., 2014b; Lebaron et al., 2016). Biochemical studies have shown that these mutations allow the re-interaction of VPg with the resistant eIF4E allele, allowing potyviruses to resume the use of the corresponding eIF4E isoform *in planta* as a susceptibility factor (Charron et al., 2008; Truniger and Aranda, 2009; Perez et al., 2012).

The appearance of mutations in *VPg* that allow the overcoming of resistance represents potyvirus evolution, which necessitates viral multiplication in the cells of resistant plants. It was discovered that low level, asymptomatic accumulation of potyviruses can occur in resistant plants (Acosta-Leal and Xiong, 2008; Montarry et al., 2011). This phenomenon is also known as subliminal infection (Acosta-Leal and Xiong, 2008). Although subliminal infections do not cause significant damage to the plant, viral multiplication allows for the emergence of mutations, some of which will lead to changes in VPg and its re-interaction with resistant eIF4E alleles (Montarry et al., 2011). Asymptomatic accumulation of potyviruses in plant tissues, which enables their evolution, has been shown to be the major determinant of the RB (Quenouille et al., 2013; Moury et al., 2016).

Faint multiplication in plants with resistant eIF4E alleles suggests that viruses can use these alleles for multiplication, although with very low efficiency, as indicated by a low infection level. Some natural eIF4E alleles, such as pepper *pvr1* or *pvr2^2^*, appear to be capable of completely blocking viral multiplication at the cellular level (Deom et al., 1997; Murphy et al., 1998) and providing extremely durable resistance (van Schie and Takken, 2014). Seemingly, these alleles are hardly used by potyviruses, even for low-level multiplication. In contrast, other alleles, such as *pvr2^3^* or *pvr2^1^*, which allow for subliminal infection (Arroyo et al., 1996; Montarry et al., 2011), are less durable (Ben Khalifa et al., 2012; Moury et al., 2014a; Moury et al., 2014b).

### 3.3 Inactivation of a single eIF4E isoform often does not lead to durable resistance

In all studied cases, potyviruses overcame resistance based on natural polymorphisms in certain eIF4E isoforms *via* re-interaction with this isoform, which was made possible by acquiring mutations in *VPg* (Charron et al., 2008; Perez et al., 2012; Lebaron et al., 2016). Thus, the obvious way to develop plants resistant to potyviruses is to inactivate this eIF4E isoform, depriving the virus of this crucial host factor. As mentioned above, plants with an inactivated single eIF4E isoform are often viable and do not exhibit severe abnormalities because of the partial redundancy between eIF4E isoforms.

To date, resistance to diverse potyviruses has been achieved in different plants by inactivating the eIF4E isoform (Duprat et al., 2002; Sato et al., 2005; Piron et al., 2010; Julio et al., 2015; Chandrasekaran et al., 2016; Pechar et al., 2022). However, in many cases, a fraction of plants with inactivated eIF4E eventually develop disease, although at a slower rate than the wild-type plants, indicating the RB process (Duprat et al., 2002; Julio et al., 2015; Chandrasekaran et al., 2016; Pechar et al., 2022). Therefore, rather than providing complete immunity, inactivating the single eIF4E isoform often provides only a nondurable resistance to potyviruses that use this isoform as a susceptibility factor.

### 3.4 Many potyviruses can use alternative eIF4E isoform variants as susceptibility factors

Potyviruses require eIF4E to perform various functions related to translation as well as other stages of their life cycle. This means that potyviruses have to use another factor in plants with inactivated eIF4E isoform. Viruses may bypass its absence by using either another eIF4E isoform or non-eIF4E cell factors. Although the latter cannot be excluded (Gallie, 2001; Khan et al., 2008), the main way to bypass the lack of the required eIF4E isoform is to use an alternative variant of the eIF4E isoform. This has been proven by the fact that plants with simultaneous inactivation of two eIF4E isoforms often demonstrate immunity to one or several potyviruses. *Arabidopsis* plants lacking both eIF4E1 and eIF(iso)4E were resistant to TuMV strains that overcome resistance based on the inactivation of eIF(iso)4E alone (Bastet et al., 2018). In tomato, resistance to multiple viruses was achieved by inactivating both *eIF4E1* and *eIF4E2* (Gauffier et al., 2016). In tobacco, the knockout of a single *eIF4E* gene was overcome by PVY (Julio et al., 2015), whereas the inactivation of two or more eIF4E factors from multiple ones in this plant rendered tobacco almost immune to PVY (Udagawa et al., 2021; Le et al., 2022). Therefore, potyviruses overcome resistance based on the absence of a particular eIF4E isoform primarily, if not exclusively, *via* the use of another eIF4E isoform.

Usually, potyvirus must undergo adaptation to use an alternative variant of the eIF4E isoform. This is accomplished by acquiring mutations in the VPg protein, which allow VPg to interact with the new eIF4E isoform. In the tobacco–PVY pathosystem, the VPg of strains that overcame eIF4E-1 inactivation carried substitutions, most often at position 105 of this protein (Michel et al., 2019), allowing VPg to bind one of the tobacco eIF(iso)4Es (Takakura et al., 2018). In TuMV, substitutions at position 116 or 163 of VPg were required to use eIF4E1 in addition to eIF(iso)4E in *Arabidopsis* (Gallois et al., 2010; Bastet et al., 2018).

The accumulation of mutations in VPg takes time and manifests as a delay in the development of the symptoms of infection. In plants with inactivated eIF4E isoforms, subliminal infections were detected, allowing potyviral evolution (Acosta-Leal and Xiong, 2008; Acosta-Leal and Xiong, 2013). Subliminal infections indicate that these viruses can use alternative eIF4E isoforms, although inefficiently, because viral accumulation is very low. During these subliminal infections, variants of the virus could appear with mutations in VPg, allowing the virus to use an alternative eIF4E isoform more efficiently. When this happens, the resistance mediated by eIF4E inactivation is broken, the virus accumulates excessively in plant tissues, and disease symptoms will develop.

Surprisingly, at least in some cases, potyviruses can efficiently use an alternative eIF4E isoform in plants directly without any apparent adaptation. This observation has been supported by evidence from the tomato–PVY and tomato–TEV pathosystems. eIF4E1 is the susceptibility factor to these viruses in tomato because its alleles (pot-1 and pot-1^2^, with 4 and 2 amino acid substitutions, respectively) confer resistance to diverse PVY and TEV strains in different tomato species and interspecific hybrids (Ruffel et al., 2005; Gauffier et al., 2016; Lebaron et al., 2016). Strikingly, inactivation of eIF4E1 yields low, if any, resistance to these viruses, with the exception of a single PVY strain (Piron et al., 2010; Gauffier et al., 2016; Kumar et al., 2022). In tomato with inactivated eIF4E1, these viruses use eIF4E2 as a susceptibility factor because double knockout of *eIF4E1* and *eIF4E2* leads to resistance (Gauffier et al., 2016; Kumar et al., 2022). Interestingly, after infection of *eIF4E1* KO tomato, no features of RB, such as infection of only a fraction of plants or delayed development of symptoms, were observed. Accordingly, no *VPg* mutations were observed (Kumar et al., 2022). This speaks in favor of the ability of different PVY and TEV strains to use eIF4E2 in tomato directly without any adaptation. In fact, the ability of PVY and TEV VPg to physically bind to tomato eIF4E2 as well as eIF4E1 was demonstrated in a yeast-two-hybrid screen (Mazier et al., 2011).

### 3.5 Main eIF4E isoform and backup eIF4E isoform

Thus, in different plant–potyvirus systems, in addition to the “main” eIF4E isoform, which is normally used by virus as a susceptibility factor and the allelic composition of which determines resistance in nature, potyviruses can use an alternative eIF4E isoform in plants with inactivation of the “main” susceptibility isoform. According to Moury et al. (2020), this eIF4E isoform can be characterized as a backup susceptibility factor. In the next part of this review, the following terms will be used: main susceptibility isoform, or main eIF4E isoform, and backup susceptibility isoform, or backup eIF4E isoform. Potyviruses normally use the main eIF4E isoform to infect the plant, but in its absence, they use the backup eIF4E isoform. The “main” and “backup” designations do not reflect their importance to normal cell physiology and are only used in the context of the specific plant–potyvirus interaction.

Plants typically have a single primary and a single backup eIF4E because inactivation of two eIF4E genes often provides robust resistance, in some cases to multiple potyviruses (Gauffier et al., 2016; Bastet et al., 2018; Udagawa et al., 2021). This is most likely due to potyviruses limited adaptability as they cannot adapt to use any eIF4E isoform in plant. However, some TEV and PVY strains were shown to partially retain the ability to multiplicate in tomato plants with inactivation of two isoforms (eIF4E1 and eIF4E2), although the level of infection was severely reduced (Gauffier et al., 2016; Kumar et al., 2022). Some potyviruses lack the ability to use the backup eIF4E isoform because the inactivation of the main eIF4E isoform provides a resistance that was not broken down (Piron et al., 2010). It is unclear whether this is due to the virus’s complete inability to adapt to another eIF4E isoform or to experimental conditions such as insufficient infection pressure or sample size. Interestingly, the ability of different strains of the same potyvirus to use the backup eIF4E isoform was shown to differ (Piron et al., 2010; Atarashi et al., 2020).

In some cases, inactivating the main eIF4E isoform did not suppress viral multiplication within cells but restricted its spread in plant tissues (Contreras-Paredes et al., 2013; Julio et al., 2015). Alternatively, potyviral translation rather than replication could be impaired (Kumar et al., 2022). This suggests that potyviruses can use alternative eIF4E factors for some but not all eIF4E-mediated activities in the viral proliferation cycle.

When potyviruses adapt to use the backup eIF4E isoform, they retain the ability to interact with the main eIF4E isoform. However, the efficiency of their application may vary. The accumulation of TuMV RB strains in *Arabidopsis* was a few-fold lower in plants with an inactivated main eIF(iso)4E isoform than in WT plants (Bastet et al., 2018). This implies that the backup eIF4E isoform is suboptimal for TuMV, even for RB strains with mutations in *VPg*. In tomato, the main eIF4E1 supports PVY multiplication more efficiently compared to the backup eIF4E2 (Kumar et al., 2022). Conversely, an RB strain of PVY in tobacco uses the backup isoform, i.e., one of the tobacco eIF(iso)4E factors, more efficiently than it uses the main isoform eIF4E-1 (Takakura et al., 2018; Udagawa et al., 2021).

### 3.6 The use of backup eIF4E isoforms as susceptibility factors is hindered in plants with functional resistant alleles of the main eIF4E isoform

In diverse plant–potyvirus pathosystems, the ability of the potyvirus to infect the plant using a backup eIF4E isoform has been demonstrated. Therefore, it may appear surprising that recessive resistance to potyviruses *via* an eIF4E-mediated mechanism is so widespread. Apparently, to achieve such resistance, it is necessary to modify not only the main eIF4E isoform but also the backup eIF4E isoform; otherwise, potyvirus will overcome the resistance by using the latter.

However, these findings contradict the observation that, in the vast majority of cases, natural eIF4E-mediated resistance was determined by the allelic composition of a single gene (**Table 1**), implying that the virus depends on a single eIF4E isoform to infect the plant. The widespread use of such genes in agriculture indicates that the level of resistance provided by them is sufficient for their practical application. Moreover, sometimes single gene *eIF4E*-mediated resistance can be extremely durable and effective against various potyviruses (van Schie and Takken, 2014; Moury et al., 2014a; Moury et al., 2014b).

One distinguishing feature of the majority of natural resistant *eIF4E* genes is that they are generally not null alleles but encode apparently functional proteins. A few amino acid substitutions in these proteins prevent their use by viruses but do not usually inhibit their function as translation initiation factors. This was confirmed by the ability of the proteins encoded by resistance *eIF4E* alleles to perform intrinsic eIF4E functions, such as binding to the cap analog *in vitro* and initiating translation in yeast cells lacking their own eIF4E (Kang et al., 2005; Charron et al., 2008; Gauffier et al., 2016).

Despite the presence of backup eIF4E isoforms in plants, the resistance conferred by natural eIF4E proteins with point mutations is overcome through re-interaction with them. In tomato, the *pot-1^2^* allele of eIF4E1 conferred a low but significant level of resistance to the PVY strain N605, although it could be overcome by single or double mutations in VPg (Lebaron et al., 2016). It is uncertain why PVY must overcome the resistance conferred by *pot-1^2^* by accumulating mutations in VPg, despite the fact that tomato contains eIF4E2, which could be directly used as a susceptibility factor by PVY-N605 without any apparent adaptation (Lebaron et al., 2016; Kumar et al., 2022). In pepper, the overcoming of resistant eIF4E1 alleles by PVY and TEV also occurs by the re-acquisition of the ability to use these “resistant” proteins. For some pepper *eIF4E1* alleles, it requires multiple VPg mutations, is hardly achieved in laboratory studies, and is not observed in the field (Ayme et al., 2007; Fabre et al., 2012; Ben Khalifa et al., 2012). Concomitantly, the VPgs of PVY and TEV were able to directly bind to pepper eIF4E2 in yeast two-hybrid screening without any mutations (Gauffier et al., 2016). However, for some reason, PVY and TEV are unable to use pepper eIF4E2 as a susceptibility factor *in planta*.

These observations led to the conclusion that natural resistant alleles of the main eIF4E isoform, which encode a functional protein with point modifications, are not just passive nonparticipants in plant–potyvirus interaction. They repress the use of alternative eIF4E isoforms as susceptibility factors (Gauffier et al., 2016; Bastet et al., 2017). Unlike functional eIF4E alleles, null alleles do not have such a repressive effect, and the resistance they impart can be relatively easily overcome by the viruses by using the backup eIF4E isoform. Hence, functional resistant alleles of the main eIF4E isoform can somehow modulate the functionality of other eIF4E isoforms as susceptibility factors. This conclusion contradicts the established view of eIF4E-mediated resistance being recessive, which requires the lack of susceptibility factors in the plant. Rather, the eIF4E isoform can inhibit the use of the other eIF4E isoforms as susceptibility factors by the virus. Because the different isoforms are encoded by nonallelic genes, it does not represent dominance; instead, it indicates an epistatic interaction between them.

#### 3.6.1 eIF4E isoforms mutually suppress each other’s accumulation

It is obvious that the effectiveness of the binding of two proteins, e.g., eIF4E and VPg, will depend on their concentrations. Accordingly, the efficiency of the use of the eIF4E isoform by potyviruses depends not only on the affinity of its binding to potyviral VPg but also on the intracellular concentration of this isoform. Reducing the concentration of eIF4E in plant cells will lead to a lower efficiency of its use by the virus, whereas its increase will have the opposite effect. In fact, increased eIF4E levels promote potyviral accumulation (Schaad et al., 2000; Nicaise et al., 2003; Hafrén et al., 2013), whereas eIF4E silencing results in resistance or at least significantly suppressed potyviral accumulation in plant tissues (Hwang et al., 2009; Eskelin et al., 2011; Mazier et al., 2011; Takakura et al., 2018; Chen et al., 2022a). Artificial silencing rarely results in complete gene repression; however, the decrease in eIF4E concentration was sufficient to achieve resistance.

**Figure 2:**
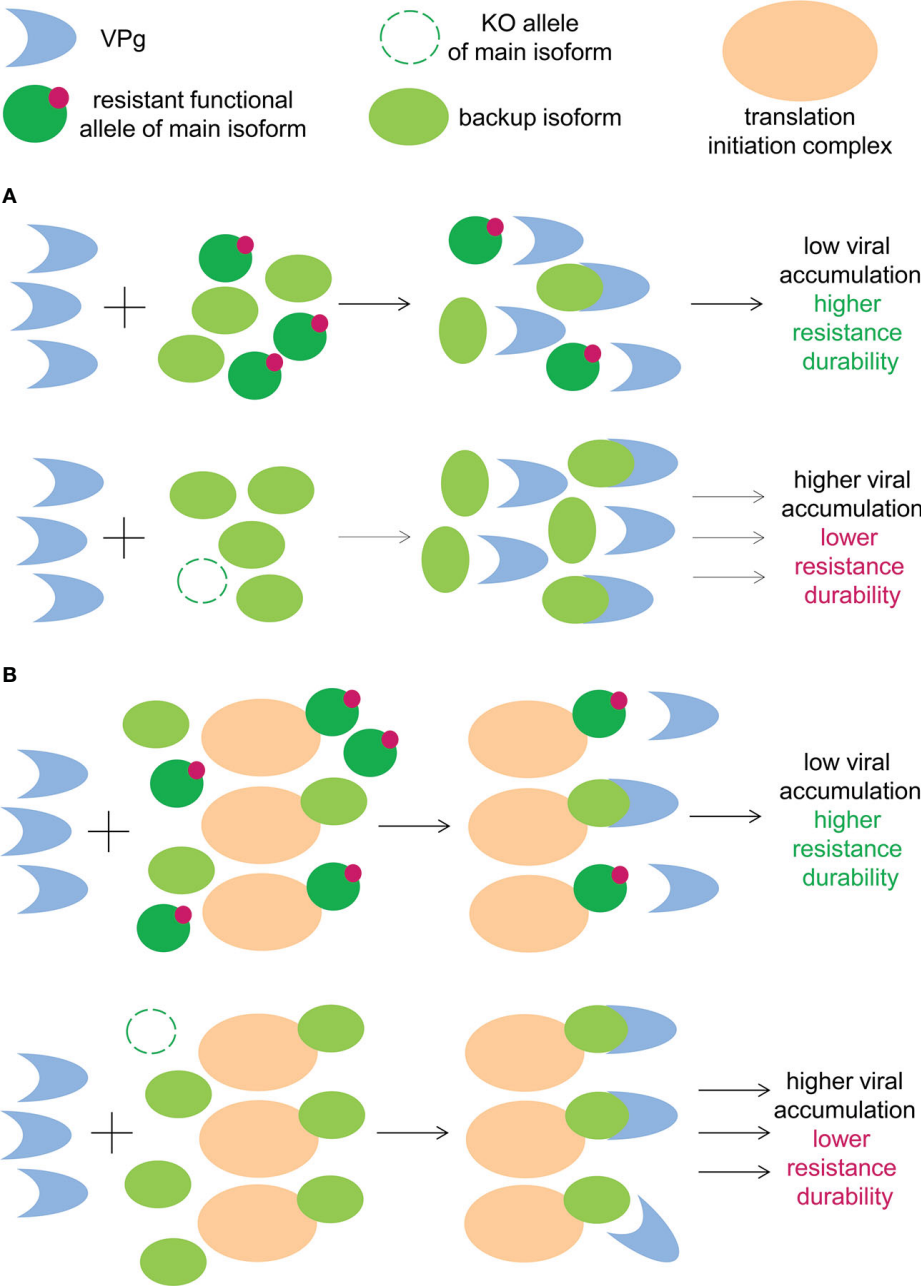
**(A)** Functional resistant allele of main eIF4E isoforms downregulates the cellular concentration of the backup eIF4E isoform, leading to reduced viral accumulation. **(B)** The functional resistant allele of the main eIF4E isoform competes with the backup eIF4E isoform for the cellular translation apparatus, leading to reduced viral accumulation.

Do the eIF4E isoforms regulate each other’s accumulation of in plant cells? As per the abovementioned information, the answer is yes. In *Arabidopsis* and tobacco, the inactivation of eIF(iso)4E led to an increase in eIF4E accumulation, although the inverse was not true (Duprat et al., 2002; Combe et al., 2005; Zafirov et al., 2021). In tomato, eIF4E1 knockout resulted in an increase in eIF4E2 levels (Gauffier et al., 2016). In the context of potyviral infection, knockout of the main eIF4E isoform will result in the compensatory overaccumulation of the remaining eIF4E isoforms in plant cells. Consequently, higher concentration of the backup eIF4E isoform will facilitate its binding to potyviral VPg and promote its use as a susceptibility factor by virus (**Figure 2A**). Conversely, in the presence of a functional resistant allele of the main eIF4E isoform, a compensatory increase in the backup eIF4E isoform concentration does not occur, which impedes its use by the virus. Tomato genotypes with a resistant eIF4E1 allele and decreased eIF4E2 concentration exhibited robust resistance to different strains of PVY and TEV, which used eIF4E2 as a backup susceptibility factor (Gauffier et al., 2016). In contrast, tomato plants with inactivated eIF4E1 accompanied by a compensatory increase in eIF4E2 concentration were susceptible to these viruses (Gauffier et al., 2016; Kumar et al., 2022).

#### 3.6.2 eIF4E isoforms are likely to compete for translation apparatus

The initiation of translation requires not only eIF4E but the entire translation initiation complex. Therefore, one possible mechanism underlying the mutual effect of the eIF4E isoforms on the availability to viruses is competition for the remaining elements of the translation initiation complex. In *Arabidopsis*, the inactivation of eIF(iso)4E increased the eIF4E concentration the polysomal actively translated fraction (Duprat et al., 2002). The functional resistant allele of the main eIF4E isoform competes with other isoforms for the translation apparatus, which reduces the efficiency of mRNA translation associated with other eIF4E isoforms, including potyviral mRNAs associated with the backup eIF4E isoform (**Figure 2B**). In plants that lack the main eIF4E isoform, this type of competition is absent; this facilitates the translation of viral mRNAs through alternative eIF4E variants. Considering that the potyviral interaction with backup eIF4E isoform is suboptimal, this quantitative increase or decrease in translational efficiency may lead to a qualitative difference in the infection course, i.e., susceptibility or resistance. Potentially, the decreased accumulation and delayed propagation of viruses in plant tissues affords the plants sufficient time to effectively develop diverse antiviral defense mechanisms (Acosta-Leal and Xiong, 2013; Contreras-Paredes et al., 2013; Nicaise, 2014). Although this was not directly observed, different studies have reported in favor of the existence and significance of the competitive effects between eIF4E isoforms in the context of plant–potyvirus interactions (Kang et al., 2007; Michel et al., 2019; Zafirov et al., 2021).

### 3.7 The ability to suppress each other as factors of susceptibility to potyviruses could be common for different eIF4E isoforms in plants

Previously in this review, it was discussed how the functional resistant alleles of the main eIF4E isoform could hamper the use of alternative eIF4E isoforms by the viruses. However, the mutual regulation of the concentration and competition effects should, to a certain extent, exist not only for the main and backup eIF4E isoforms but for all eIF4E factors in plant cells. Accordingly, the presence of each eIF4E factor that potyvirus could not use should promote resistance by decreasing the availability of “susceptible” eIF4E isoforms to this virus to some extent.

To date, evidence has confirmed this assumption in different plant–potyvirus pathosystems. In pepper, the durability of PVY resistance mediated by eIF4E1 alleles substantially decreased with the inactivation of eIF(iso)4E. The gene encoding eIF(iso)4E was the major QTL that defined resistance durability, and the existence of a functional eIF(iso)4E in pepper increased PVY resistance (Quenouille et al., 2014, 2016). Because PVY on pepper overcomes resistant eIF4E1 alleles by re-interacting with them through the accumulating mutations in VPg, the presence of functional eIF(iso)4E in pepper cells obviously suppressed this re-interaction. eIF(iso)4E, which is not used by PVY as a susceptibility factor, apparently downregulated eIF4E1 concentration and/or competed with it for the cellular translational machinery. This hampers the use of resistant eIF4E1 allelic variants by PVY, which is anyway inefficient, thus suppressing subliminal infections and increasing resistance durability.

In tobacco, the durability of PVY resistance conferred by *eIF4E-1* deletion depends heavily on an additional genetic locus that contains single or multiple copies of *eIF4E-2* (Michel et al., 2019). Although *eIF4E-2* is phylogenetically referred to as an eIF4E isoform (**Figure 1**), similar to *eIF4E-1*, it was inherited by tobacco from PVY-resistant *N. tomentosiformis* and is not used by this virus as a susceptibility factor. An excellent link was identified between the *eIF4E-2* expression levels in tobacco and the durability of PVY resistance (Michel et al., 2019); therefore, the increased accumulation of eIF4E-2 in tobacco cells qualitatively impeded RB. As RB in plants with an inactivated main eIF4E isoform occurs through the use of the backup eIF4E isoform, the increased concentration of eIF4E-2 appeared to decrease the efficiency of its use by PVY. Michel et al. (2019) suggested that the genome segment containing and expressing multiple *eIF4E-2* copies corresponds to the previously identified *va2* locus of tobacco PVY resistance. In combination with the inactivation of the main susceptibility factor, eIF4E-1, the *va2* locus greatly enhances resistance durability. Moreover, *va2* is able to confer partial PVY resistance by itself (Acosta-Leal and Xiong, 2008; Lacroix et al., 2011). The presence of *va2* reduces the accumulation of PVY in tobacco cells (Acosta-Leal and Xiong, 2008), possibly because of a decrease in viral translation and replication.

**Figure 3:**
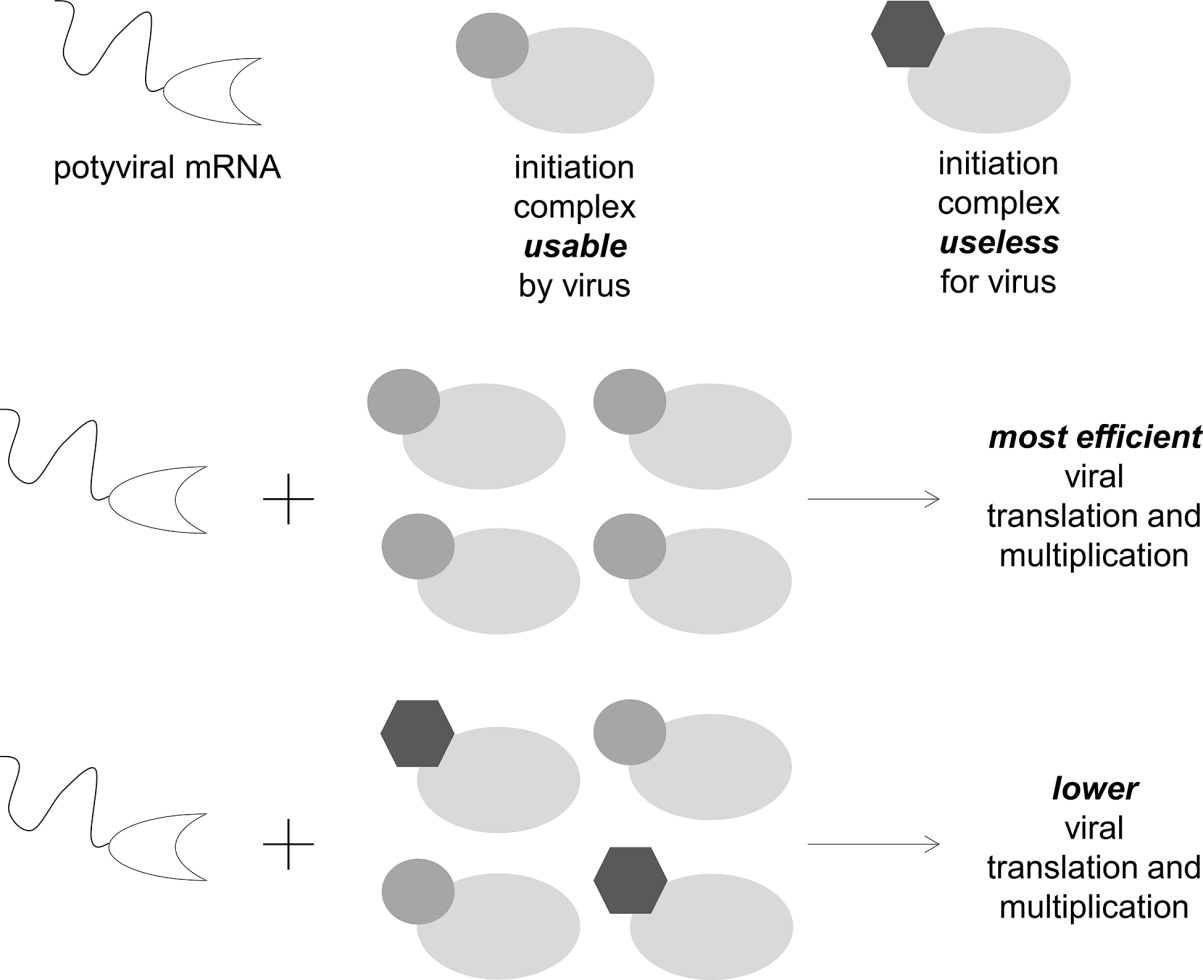
eIF4E isoforms that cannot be used by viruses can decrease their accumulation in plant cells by the mutual regulation of concentration and competition effects.

Taken together, these results suggest that eIF4E isoforms that are not directly involved in the plant–potyvirus interaction could affect its outcome. These isoforms could indirectly increase eIF4E-mediated resistance by reducing the efficiency of the use of the main or backup eIF4E isoforms by potyviruses. Therefore, they in some way “dilute” the cell translation complexes that are suitable for the virus, thereby decreasing its multiplication efficiency (**Figure 3**). Although this effect is unlikely to be adequate to afford plant resistant by itself, the aforementioned examples indicated that it could be significant in the context of resistance durability.

## 4 Search for the main eIF4E isoform: Functional tests, biodiversity analysis, or both

The main susceptibility eIF4E isoform is also the main resistance isoform because the resistant alleles of this isoform do not support infection and hinder the use of backup eIF4E isoforms by the virus, thus exerting an epistatic effect on them as a susceptibility factors. Considering its importance, what approaches can be used to identify the eIF4E isoform, which is the main isoform for a given potyvirus? This task is most simple in plants, for which there are known eIF4E alleles that confer recessive resistance to the virus of interest (**Table 1**). However, these alleles for many important crops that are heavily damaged by potyviruses, such as potato, soybean, cassava, or plum, are unknown.

Because the interaction of eIF4E with viral proteins, particularly VPg, is required for potyvirus multiplication, the most obvious way to determine whether the isoform can be used by potyviruses is to analyze the protein–protein interactions. A match between the binding of VPg with eIF4E in model systems (mainly yeast two-hybrid screening) and the ability of this eIF4E to sustain potyviral infection has been proven numerous times (Léonard et al., 2000; Kang et al., 2005; Charron et al., 2008; Mazier et al., 2011; Perez et al., 2012; Takakura et al., 2018). However, in some cases, discrepancies, or at least incomplete consistencies, were observed between the eIF4E isoforms that interacted with VPg on protein–protein interaction screening and isoforms used by the potyviruses *in planta* as susceptibility factors (Gao et al., 2004; Gallois et al., 2010; Estevan et al., 2014; Moury et al., 2020). Apparently, these discrepancies are explained by the fact that model systems are heterologous and do not consider different factors of plant cells, such as the relative concentrations of each isoform and the competitive effects between them, as a single eIF4E protein is studied in heterologous systems.

Another straightforward approach, i.e., complementation of potyviral infection by the eIF4E isoform expression in resistant plants, could also provide unclear results. *B. rapa* contains multiple factors belonging to both eIF(iso)4E and eIF4E isoforms. In this plant, TuMV exclusively uses some eIF(iso)4E variants, but not any of the eIF4Es, as susceptibility factors (Rusholme et al., 2007; Qian et al., 2013; Nellist et al., 2014). However, transgenic overexpression of different *B. rapa* eIF4E factors complement TuMV infection in *Arabidopsis* plants with inactivated AteIF(iso)4E (Jenner et al., 2010). This is apparently explained by the use of the strong 35S promoter to express eIF4E, leading to its overaccumulation in plant cells and facilitating its use by potyvirus.

The reverse genetics approach can also be used to determine the role of eIF4E isoforms as susceptibility factors. If eIF4E isoform inactivation confers resistance, this proves its role as a main susceptibility factor to a given potyvirus, even if RB occurs eventually. However, in some pathosystems, such as tomato with TEV and different PVY strains, the inactivation of the main isoform (eIF4E1 in these cases) did not result in any significant resistance (Piron et al., 2010; Gauffier et al., 2016). In potato, eIF4E1 inactivation reduced PVY multiplication (Lucioli et al., 2022). Notably, the suppression of nCBP in potato cells has a similar effect to one PVY strain (Chen et al., 2022a). Although these data strongly suggest that at least some PVY strain could unexpectedly use multiple and least-similar eIF4E isoforms in potato, the inactivation of a single eIF4E isoform would likely disturb some regulatory mechanism between different eIF4E isoforms. Consequently, the effect of eIF4E inactivation on susceptibility to potyviruses should be interpreted with some caution.

As studying the role of isoforms in plant–potyvirus interaction using direct approaches could be problematic, indirect approaches are more likely to be useful. Although eIF4E are highly conserved proteins, viral infection in different plants strongly promotes diversifying selection of eIF4E sequences, which leads to the appearance of point mutations (Cavatorta et al., 2008; Hofinger et al., 2011; Konečná et al., 2014; Moury et al., 2014a; Moury et al., 2014b; Poulicard et al., 2016). For example, in pepper, more than two dozen allelic variants of the eIF4E1 isoform, which is the susceptibility factor to different potyviruses (**Table 1**), were discovered (Charron et al., 2008; Ibiza et al., 2010; Jeong et al., 2012; Moury et al., 2014a; Moury et al., 2014b; Poulicard et al., 2016). Moreover, multiple alleles were identified for pepper eIF(iso)4E (Ibiza et al., 2010), which is also used by some potyviruses as a susceptibility factor (Hwang et al., 2009; Rubio et al., 2009). The selection pressure resulting from viral infection manifests itself in the form of predominance of nonsynonymous substitutions over synonymous ones, mainly in the eIF4E–VPg interaction interface (Charron et al., 2008; Poulicard et al., 2016). The accumulation of such substitutions in the eIF4E isoform strongly suggests that this isoform is a susceptibility factor for the most common potyvirus(es).

Interestingly, the allelic diversity of eIF4E sequences was repeatedly shown to be higher in domesticated populations compared with wild ones (Hofinger et al., 2011; Konečná et al., 2014; Poulicard et al., 2016; Yang et al., 2017). This is presumably caused by cultivation in dense monocultures, which triggers a higher viral pressure and provokes plant evolution toward resistance. According to the law of homologous series in variation (Vavilov, 1922; Nanjundiah et al., 2022), the existence of resistant eIF4E alleles is expected in diverse plants that are widely infected by potyviruses. For autopolyploid species, such as potato, where the spontaneous emergence of eIF4E-mediated resistance is relatively unlikely because of its recessive nature and the presence of several almost identical genes encoding each eIF4E isoform, accessing eIF4E biodiversity in diploid relatives could be more productive. As the occurrence frequency of eIF4E allelic variants could be low in some cases (Nieto et al., 2007), large-scale genotyping studies are warranted.

Although the polymorphisms in the eIF4E isoform suggest that it is used by viruses as a susceptibility factor, the exact virus that caused them cannot be determined using this approach. To determine whether these polymorphisms are involved in resistance to a potyvirus of interest, common methods of studying protein–protein interactions between eIF4E alleles and the VPg of this virus could be used. In such model systems, different alleles of the same isoform instead of different isoforms, are compared. Therefore, the effect of the factors that could lead to misinterpretation, e.g., interplay between isoforms, could be diminished.

## 5 From biology to biotechnology

Knowledge of the interaction between eIF4E isoforms in plant–virus interactions is required to develop optimal strategies to obtain plants with robust eIF4E-mediated resistance.

This resistance is hardly unimaginable without modifying the main eIF4E isoform. Fortunately, there is only a single main isoform in most plant–virus interactions that facilitates the introgression of this type of resistance. After the identification of the main eIF4E isoform, the best option is to introduce point amino acid changes which will preclude its binding to the viral VPg without having significant negative impacts on eIF4E functionality as a translation initiation factor of cellular mRNAs (Bastet et al., 2017). This will allow us to avoid the possible pleiotropic effects of the loss of eIF4E function as well as to maintain the repressive epistatic effect of the main isoform on other eIF4E isoforms as susceptibility factors *in planta*.

This task is most simple if the resistant alleles of the main eIF4E isoform in the plant of interest, or at least in its close relatives, are known. Resistance-conferring mutations could be introduced in the main eIF4E isoform of a susceptible cultivar through hybridization or more modern approaches, such as genome editing. However, the use of the latter is significantly hampered by the fact that naturally resistant eIF4E alleles almost always have multiple substitutions relative to susceptibility ones. Moreover, these substitutions are often present at a distance of a few to a few dozen nucleotides (Nicaise et al., 2003; Gao et al., 2004; Charron et al., 2008). Genome editing approaches, which are best suited for the introduction of such mutation combinations in plant genomes (prime editing and editing using homology-directed repair), currently have low efficiency in plants (Čermák, 2021). The use of traditional selection methods to introduce resistant alleles is more developed approach; however, it is hampered by other considerations, for example, low compatibility or cosegregation with undesirable traits.

Natural eIF4E-mediated resistance has not been discovered in many important crops; therefore, resistant eIF4E alleles are unavailable. In such cases, resistant alleles could be artificially created by introducing substitutions that are homologous to resistance-associated mutations in eIF4Es of other plants (Yeam et al., 2007; Bastet et al., 2019) or through using protein engineering approaches (German-Retana et al., 2008; Ashby et al., 2011). However, the effect of such mutations may not be conserved after transferring into eIF4E of the other plant (Cavatorta et al., 2011; Bastet et al., 2019). Moreover, the consequences of such modifications on eIF4E functionality in plants are hardly predictable, especially in the case of artificially designed substitutions.

An alternative variant of modification, which is not accompanied by the aforementioned difficulties, is the knockout of the main eIF4E isoform. It is easier to inactivate the protein than to precisely modify it while retaining its functionality in plant cells but not to the virus. Moreover, novel plant genome editing technologies with limited success in introducing precise substitutions, especially multiple ones, are much more efficient in inactivating genes through the introduction of indel mutations or deletions.

Resistance to potyviruses based on the inactivation of the main eIF4E isoform is currently used in agriculture. Several widely grown tobacco varieties lack the eIF4E-1 factor because of a large genomic deletion or point nonsense mutations and are resistant to PVY and TVMV (Nicolas et al., 1996; Julio et al., 2015; Lin et al., 2021). Also, some varieties of *B. rapa* display resistance to TuMV because of the miss-splicing of one of the *eIF(iso)4E* genes (eIF(iso)4E.a) (Nellist et al., 2014). Although the PVY isolates that overcome this resistance in tobacco are common, inactivation *eIF4E-1* lowers the probability of a tobacco plant being infected by PVY in the field (Lacroix et al., 2010). In *B. rapa*, eIF4E-mediated resistance persists and remains effective against multiple TuMV strains (Nellist et al., 2014).

A major potential concern associated with eIF4E inactivation is the negative pleiotropic effects on plant growth and yield, which could be indistinguishable in the laboratory but can have a significant impact under stressful field conditions. Apparently, resistance based on eIF4E inactivation is most suitable for species that carry multiple eIF4E factors belonging to different eIF4E isoforms. Tobacco has six eIF4Es and two eIF(iso)4Es (Julio et al., 2015), whereas *B. rapa* has three eIF(iso)4Es and three eIF4Es (Jenner et al., 2010) (or even more (Kim et al., 2013; Qian et al., 2013)). Inactivation of the single main eIF4E or even of main and backup eIF4Es in plants with multiple isoforms will likely not have any considerable pleiotropic effects on plant growth and development. This allows the use of cultivars with inactivated eIF4E in agriculture without significant yield penalties.

The second major concern associated with resistance mediated by nonfunctional alleles of the single (main) eIF4E isoform is its low robustness owing to the ability of the virus to switch to another (backup) eIF4E isoform (Bastet et al., 2017). To improve resistance durability, this backup isoform needs to be modified. In tobacco, PVY resistance durability mediated by eIF4E-1 inactivation could be markedly improved by inactivating the backup susceptibility isoform (Udagawa et al., 2021; Le et al., 2022). In *B. rapa*, durable broad-spectrum resistance to TuMV besides eIF(iso)4E.a inactivation also required a certain resistant allele (named *ConTR01*) of eIF(iso)4E.c, which is apparently used by TuMV as backup susceptibility factor (Rusholme et al., 2007; Nellist et al., 2014).

Another possible complementary approach to improve resistance durability is the combination of the resistant eIF4E allele with additional resistance-associated QTLs (Naderpour et al., 2010; Quenouille et al., 2013, 2014), which could sometimes be eIF4E. The high significance of such eIF4E-QTLs for resistance to potyviruses has been demonstrated in pepper (Quenouille et al., 2014, 2016) and tobacco (Acosta-Leal and Xiong, 2008; Lacroix et al., 2011; Michel et al., 2019) for PVY and in plum for the plum pox virus (Decroocq et al., 2005; Marandel et al., 2009a, 2009b). Apparently, they represent eIF4E isoforms that could not be used by the virus and decreased its accumulation in plant cells through the abovementioned mechanisms, i.e., downregulating the concentration of the main and backup eIF4E isoforms and competing with them for the cell translational apparatus. It seems appropriate to conduct studies that can thoroughly search for such eIF4Es that quantitatively enhance resistance to potyviruses while considering not only amino acid sequences but also the expression levels. It is expected that plants with increased accumulation of different eIF4Es that cannot be used by potyvirus represent a good genetic background to develop varieties with robust resistance.

## 6 Conclusions

Although this review mainly discussed eIF4E-mediated resistance to potyviruses, it is applicable to viruses from other groups as well. Although not studied as thoroughly, this resistance is likely to follow the same rules. Therefore, understanding the interaction between eIF4E isoforms in plant–potyvirus interactions could also be used to develop resistance to other viruses, especially to those systematically close to potyviruses.

A very interesting, albeit almost unstudied, question is *why* viruses use one or the other eIF4E isoform as a susceptibility factor. Currently, only assumptions can be made regarding this. To function as a susceptibility factor, the eIF4E isoform should be available to potyviruses in sufficient quantity. Most potyviruses use eIF4E as the main susceptibility factor, which is presumably explained by the generally more ubiquitous expression. However, in *Arabidopsis*, for instance, the majority of the studied potyviruses use eIF(iso)4E as a susceptibility factor (Sato et al., 2005). This could possibly be explained by the increased accumulation of the isoform at certain developmental stages, during which natural infection predominantly occurs. Interestingly, in *Arabidopsis*, eIF(iso)4E is more abundant in young tissues (Rodriguez et al., 1998).

Even the functions of plant eIF4Es on their own as susceptibility factors to viruses are still far from being well understood. The most investigated topic is the participation of eIF4E in viral translation and replication. However, eIF4E-mediated resistance sometimes acts on different levels, e.g., blocking the cell-to-cell or systemic movement of the virus. Although possible mechanisms of eIF4E involvement in these processes have been proposed a long time ago (Lellis et al., 2002), this subject is currently underexplored.

Considering the variety of eIF4E isoforms that can be used as susceptibility factors by different potyviruses in different plants, it is interesting to know that specific potyvirus generally rely on a single eIF4E isoform in a specific plant. Apparently, different isoforms have different interaction interfaces with VPg, which hampers the ability of the potyvirus to efficiently use multiple eIF4E isoforms. Furthermore, potyviruses have to use eIF4E efficiently to allow its rapid distribution in plants before the plant mounts antiviral defenses, which will lead to its recovery from infection that was observed in some studies (Masuta et al., 1999; Ibiza et al., 2010; Chandrasekaran et al., 2016). This necessitates viral specialization on the single eIF4E isoform, thus allowing single-locus eIF4E-mediated resistance and restricting the number of possible backup eIF4E isoforms.

## Author contributions

NZ wrote the manuscript and prepared the figures. VT wrote and edited the manuscript. Both authors have reviewed and approved the manuscript. All authors contributed to the article and approved the submitted version.
